# Undiagnosed glaucoma and its risk factors in the Yinzhou community-based glaucoma screening program

**DOI:** 10.3389/fpubh.2025.1671225

**Published:** 2026-01-12

**Authors:** Qi Chen, Yang Cao, Mengtian Zhou, Xuanli Zheng, Junhong Jiang, Yize Chen, Di Song, Peng Shen, Hongbo Lin, Juntao Zhang, Qinkang Lu, Yuanbo Liang

**Affiliations:** 1 Henan Eye Hospital, Henan Eye Institution, Henan Provincial People's Hospital, Zhengzhou University People's Hospital, Zhengzhou, Henan, China; 2 Department of Ophthalmology, First Hospital of Shanxi Medical University, Taiyuan, China; 3 Department of Ophthalmology, Eye Valley Super Eye Hospital, Wenzhou, China; 4 National Clinical Research Center for Ocular Diseases, Eye Hospital, Wenzhou Medical University, Wenzhou, China; 5 Department of Ophthalmology, Shanghai General Hospital, Shanghai Jiao Tong University School of Medicine, National Clinical Research Center for Eye Diseases, Shanghai, China; 6 Department of Ophthalmology, The First People’s Hospital of Huzhou, The First Affiliated Hospital of Huzhou Teacher College, Huzhou, China; 7 Yinzhou District Center for Disease Control and Prevention, Ningbo, China; 8 The Affiliated People’s Hospital of Ningbo University, Ningbo, China; 9 Glaucoma Research Institute, Wenzhou Medical University, Wenzhou, China

**Keywords:** glaucoma screening, undiagnosed glaucoma, risk factors, electronic medical record, health informatics

## Abstract

**Purpose:**

To analyze the risk factors associated with previously undiagnosed primary glaucoma.

**Methods:**

This cross-sectional study was conducted among residents aged 50 years and older in primary health care centers in Yinzhou District, China, from November 26, 2020, to December 3, 2021. Participants underwent various screening procedures. Patients with suspected glaucoma and other ocular abnormalities were referred to Yinzhou People’s Hospital for further examination, diagnosis and treatment. We integrated ophthalmic screening data with the Yinzhou Regional Health Information Platform (YRHIP).

**Results:**

A total of 344 patients aged 50 years or older were diagnosed with primary glaucoma, including 142 (41.3%) previously undiagnosed patients. Among the 191 patients with primary angle-closure diseases and the 153 patients with primary open-angle glaucoma, 87 (45.5%) and 55 (35.9%), respectively, were previously undiagnosed. According to the multivariable models, the factors significantly associated with decreased odds of having previously undiagnosed glaucoma at the time of screening were older age (all *p* values <0.01), worse visual acuity (PVA) (OR 0.37, 95% CI 0.18–0.76), a greater vertical cup-to-disc ratio (VCDR) (OR 0.12, 95% CI 0.02–0.99), and high-tension glaucoma (HTG) (OR 0.02, 95% CI 0.00–0.09). Compared with patients who had undergone their last visit for eye care within 1 year, patients who had last seen an eye care provider >2 years prior to the time of screening were more likely to be undiagnosed at the time of screening (all *p* values <0.05).

**Conclusion:**

Younger age, longer duration since the last eye care visit, better PVA, smaller VCDR, and normal-tension glaucoma (NTG) were all significantly associated with an increased likelihood of previously undiagnosed primary glaucoma.

## Highlights

**What is already known on this topic**

Various studies have analyzed risk factors for undiagnosed glaucoma, mainly primary open-angle glaucoma.

**What this study adds**

Longer duration since the last eye care visit, and a lower vertical cup-to-disc ratio accounts for primary angle closure diseases previously undiagnosed in the urban population of China.

**How this study might affect research, practice or policy**

Public health policies targeting populations with risk factors for previously undiagnosed glaucoma are urgently needed.

## Introduction

Glaucoma is a leading cause of irreversible blindness worldwide, and its global prevalence is 3.54% for the population aged 40 to 80 years (1). The number of glaucoma patients is increasing rapidly because of population aging and increasing in life expectancy. However, population-based epidemiological surveys have reported that at least 50% of patients with glaucoma are undiagnosed in developed countries (2–7), while more than 90% of patients are undiagnosed in developing countries (8–12). Undiagnosed glaucoma increases the risk of visual impairment, thereby adversely affecting quality of life and socioeconomic status. Early Manifest Glaucoma Trial (EMGT) reported that 62.7% of previously undiagnosed glaucoma patients had moderate to severe visual field defects (13).

Currently, undiagnosed glaucoma is detected mainly through opportunistic case-finding in clinical practice. There is no acknowledged and sustainable glaucoma screening model in China. Zhang et al. demonstrated 83.9% positive predictive value in a health examination center-based glaucoma screening model (14), while population-based glaucoma screening still has not been integrated into existing health examination systems. This lack of screening and the absence of subjective symptoms in the early stages have resulted in glaucoma remaining undetected until the onset of significant visual dysfunction. However, undiagnosed glaucoma can be prevented by early detection and management through population-based screening. Therefore, it is vital to identify risk factors associated with undiagnosed glaucoma to optimize screening programs and achieve higher case confirmation rates in community settings.

Previously, various studies have analyzed risk factors for undiagnosed glaucoma (15–20), mainly primary open-angle glaucoma (POAG). However, few studies have investigated the risk factors related to undiagnosed primary angle-closure glaucoma (PACG) (20). The risk of severe visual impairment is three times greater in patients with PACG than in those with POAG (21). China currently has the largest number of glaucoma patients in the world, and the prevalence of PACG among people of Chinese descent is 2–4 times greater than that among people of other ethnic groups (22). Therefore, there is an urgent need to investigate the risk factors for undiagnosed PACG or POAG via epidemiological studies in the Chinese population.

In this study, we integrated community-based glaucoma screening into primary health care centers in the Yinzhou district and monitored the eye care follow-up of glaucoma patients through the Yinzhou Regional Health Information Platform (YRHIP) (23). This program provides an opportunity to analyze factors associated with previously undiagnosed glaucoma using the YRHIP.

## Methods

**Figure 1:**
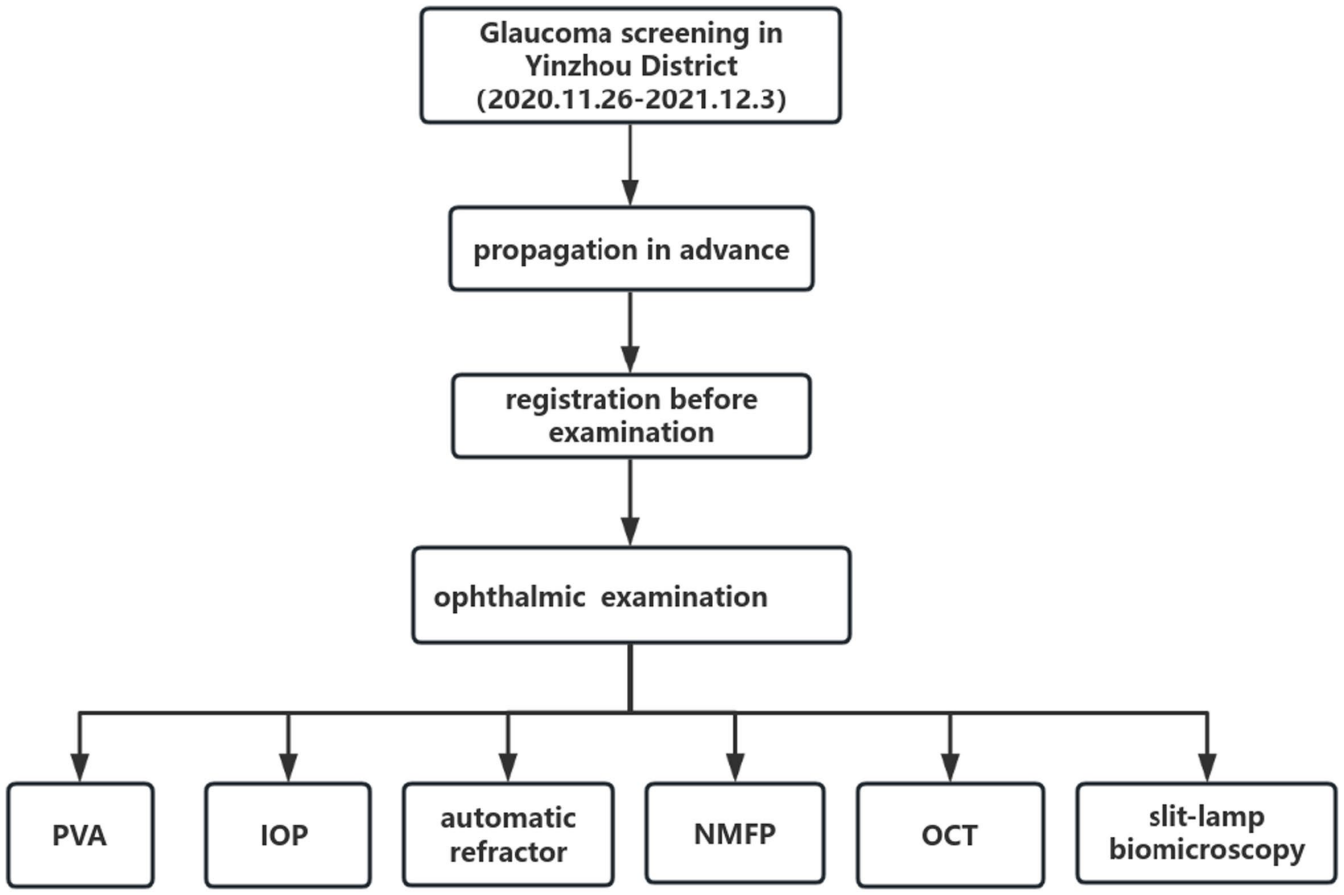
Flowchart of the Yinzhou glaucoma screening program. PVA, presenting visual acuity; IOP, intraocular pressure; NMFP, nonmydriatic fundus photography; OCT, optical coherence tomography.

This cross-sectional study was part of a randomized controlled trial (RCT) of community-based glaucoma screening in Yinzhou District. The Ethics Committee of the Eye Hospital of Wenzhou Medical University (Wenzhou, China) granted approval for this study prior to its commencement, which was conducted in accordance with the principles outlined in the Declaration of Helsinki. Written informed consent was obtained from all participants. The protocol has been published on the website^1^ with the registration number ChiCTR2200059277. In brief, we randomly selected six communities in Yinzhou District (Ningbo city, China) in which to conduct glaucoma screening. Before screening, we distributed and propagated screening activities to community staff in advance, and community workers recruited residents aged 50 and older to participate in the screening. Glaucoma screening was integrated with routine community health examinations in primary health care centers from November 26, 2020, to December 3, 2021. The screening items included registration, presenting visual acuity (PVA), intraocular pressure (IOP), automatic refractor, nonmydriatic fundus photography, optical coherence tomography (OCT) and handheld slit-lamp biomicroscopy. The glaucoma screening flowchart is shown in Figure 1.

### Patient and public involvement statement

Patients or the public were not involved in the design, conduct, or reporting or dissemination plans of our research.

### Participants

**Figure 2:**
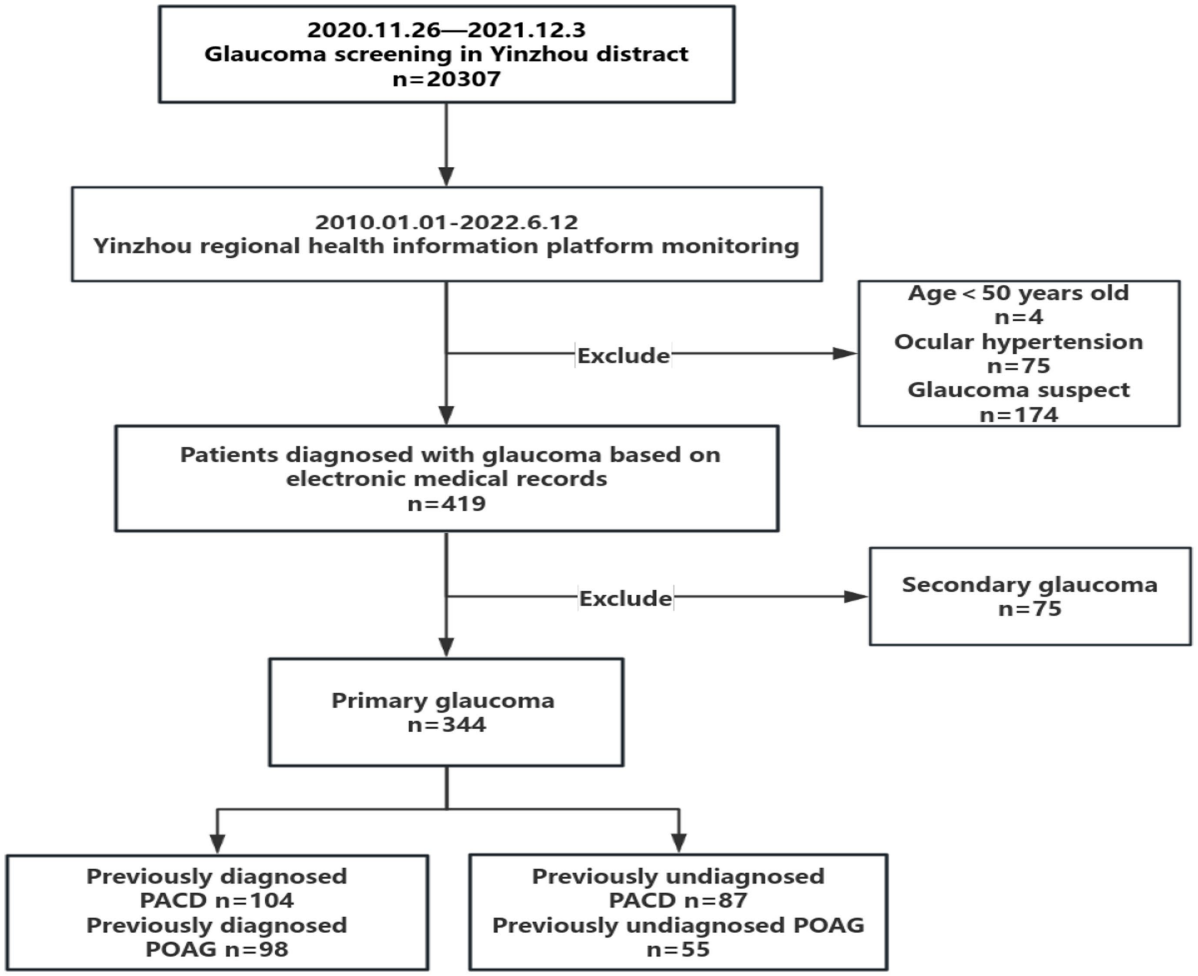
Flowchart of glaucoma monitoring through YRHIP. PACD, primary angle closure disease; POAG, primary open angle glaucoma.

The subjects included in this study were (1) individuals aged 50 years and older who underwent glaucoma screening between November 26, 2020, and December 3, 2021, and (2) individuals who had a diagnosis of primary glaucoma recorded in the YRHIP. Subjects who did not undergo any of the screening items were excluded from the study. The flowchart of participant inclusion and exclusion is shown in Figure 2.

### Community glaucoma screening at primary health care centers

From November 2020 to December 2021, a total of three professionally trained screening teams conducted glaucoma screening in primary health care centers in the Yinzhou district. Once consent was obtained, screening personnel registered the subjects, including basic information such as name, sex, and date of birth, and generated corresponding screening identification numbers to protect the subjects’ privacy.

A logarithmic visual acuity chart was used to measure the PVA of the left and right eyes successively. If the subject wore corrective glasses, the corrected vision was examined and recorded. Automatic optometry (NIDEK AR-1, Nick, Japan) was used to record the refractive status of the subjects, and IOP was measured using a noncontact tonometer (TX-20, Canon, Japan), with the average of three measurements recorded.

In the darkroom environment of primary health care centers, a trained operator took monoscopic fundus photographs using a nonmydriatic fundus camera (VISUCAM 224, Zeiss, Germany). The camera allowed for a 45-degree field, and the internal fixation point corresponded to the midpoint of the line connecting the optic disc and the macula. The photographic sequence was the right eye first, followed by the left eye. The operator selected and uploaded the optimal image for each eye onto the Eye Health Comprehensive Data Cloud Management Platform. Imaging staff in the Digital Reading Department of the Eye Hospital of Wenzhou Medical University logged into the management platform and reviewed the image data during the screening period. The quality grading and diagnosis of fundus images were evaluated by the reading staff, and fundus reading reports were uploaded at the same time. Image quality was classified as reliable, readable with both the optic disc and macula visible, and readable with only the optic disc visible or unreadable (24). The parameters of the optic disc and macula were scanned using OCT (Cirrus 5,000, Zeiss, Germany). Finally, slit-lamp biomicroscopy of the anterior segment was performed by an ophthalmologist onsite, and diagnoses were made based on ophthalmic examinations and fundus reading reports.

Suspected glaucoma (GS) (25) was diagnosed if at least one eye had any of the following features: (1) a cup:disc ratio (CDR) ≥ 0.6, CDR asymmetry ≥ 0.2, or neural rim tissue ≤ 0.1; (2) localized or diffuse retinal nerve fiber layer defects; (3) any optic disc hemorrhage; (4) “yellow” or “red” code in at least one of the quadrant maps or the clock-hour maps, excluding artifacts and segmentation errors affecting measurement reports (26); or (5) angle closure. Angle closure was diagnosed if the peripheral anterior chamber depth was <1/4 of the corneal thickness under a short vertical slit beam (27). The diagnostic criteria for other ocular abnormalities were based on the Comprehensive Adult Medical Eye Evaluation Preferred Practice Pattern Guidelines (28).

### Confirmatory eye examination

All participants diagnosed with GS or other ocular abnormalities and those with unreadable images in at least one eye were invited to the Yinzhou People’s Hospital, which served as the first tertiary hospital in the Yinzhou district and received all referrals from primary health care centers; all participants were scheduled for a complete eye examination. They could also choose other hospitals for confirmatory eye examination.

Subjects who were diagnosed with GS via community screening further underwent visual field testing (Humphrey Field Analyzer, Zeiss, Dublin, CA) free of charge at the Yinzhou People’s Hospital. Afterward, a senior glaucoma expert made a clinical diagnosis of glaucoma, GS or nonglaucoma by combining comprehensive examinations and history. Glaucoma patients were recommended for outpatient registration on the same day and underwent further examination, treatment and ophthalmic follow-up. Patients with GS were also recommended for further ophthalmic follow-up. If patients diagnosed with GS at the community screening chose to visit other hospitals, ophthalmologists also made a final diagnosis, initiated treatment and provided follow-up based on relevant examinations and patient history.

### Glaucoma definition and quality control

In this study, primary angle-closure diseases (PACDs) (29) included primary angle-closure suspect (PACS), primary angle-closure (PAC), and PACG. PACS was defined as the presence of ≥ 180^0^ appositional contacts between the peripheral iris and posterior trabecular meshwork without glaucomatous optic neuropathy (GON). PAC was defined as the presence of an occludable drainage angle ≥ 180^0^ with peripheral anterior synechiae (PAS) or elevated IOP but without secondary causes such as neovascularization, uveitis, trauma or lens-related causes. PAC accompanied by GON was classified as PACG. POAG included high-tension glaucoma (HTG) and normal-tension glaucoma (NTG). HTG was defined as the presence of an open anterior chamber angle of 360 degrees in the presence of GON and corresponding visual field damage with a 24-h peak IOP exceeding 21 mmHg, excluding secondary causes. NTG was defined as the presence of an open anterior chamber angle of 360 degrees in the presence of GON and corresponding visual field damage without 24-h peak IOP exceeding 21 mmHg, excluding secondary causes. The diagnosis of glaucoma was determined by two ophthalmologists (CQ and ZJT) with more than 6 years of diagnostic experience. If the diagnosis was inconsistent, the final diagnosis was made after discussion.

Patients who were not diagnosed with glaucoma; who had no history of glaucoma medication use, laser treatment, or surgery; or who were diagnosed with glaucoma during ophthalmic follow-up after community screening were considered to have undiagnosed glaucoma. Patients who were diagnosed with glaucoma before community screening were considered to have previously been diagnosed with glaucoma.

### Data collection

The study data included ophthalmic screening data, health examination data and electronic medical records (EMRs). We integrated ophthalmic data from the Eye Health Comprehensive Data Cloud Management Platform with patients’ EMRs from the YRHIP using unique encrypted identifiers (30). Primary glaucoma was defined in accordance with the International Classification of Diseases 10th edition (ICD-10) or diagnostic names, as shown in Table 1. The data on diagnostic names, ICD-10, diagnostic dates, and time since the last ophthalmic follow-up recorded between January 2010 and June 2022 were retrieved from EMRs. Information on age, sex, marital status, residence, body mass index (BMI), mean arterial pressure (MAP), and blood biochemical indices was extracted from health check databases. Data on the absolute spherical equivalent (ASE), PVA, IOP, and vertical cup-to-disc ratio (VCDR) were extracted from ophthalmic screening data.

**Table 1:** ICD-10 codes for primary glaucoma.

Classification/stage of primary glaucoma	ICD coding
POAG	H40.1, H40.100, H40.103
HTG	H40.1, H40.100,
NTG	H40.103
PACG	H40.2, H40.200, H40.201, H40.202, H40.203, H40.204
Acute angle closure glaucoma	H40.203
Chronic angle closure glaucoma	H40.201, H40.202, H40.204
Primary angle closure	NA
Primary angle closure suspect	H40.002

POAG, Primary open angle glaucoma; HTG, High-tension glaucoma; NTG, Normal-tension glaucoma; PACG, Primary angle closure glaucoma; PAC, Primary angle closure; PACS, Primary angle closure suspect; ICD-10, International Classification of Diseases 10th edition.

### Statistical analysis

The sample size was calculated based on a cluster RCT design; the details are provided in previous reports (24). The worse-seeing eyes of each participant were selected for analysis. Continuous variables are reported as the mean ± standard deviation, and categorical variables are described using frequency and percentage (%). Differences between groups were compared using the t test, Mann–Whitney U test, or chi–square test. Multivariate logistic regression analysis was performed to assess the risk factors associated with undiagnosed primary glaucoma, PACD or POAG. Variables with an associated *p* value less than 0.05 in the univariate analysis were included in the multivariate model. Before regression analysis, we tested for collinearity among independent variables (31) and handled missing data on the ASE and IOP through multiple imputation using linear regression models (32). Statistical analysis was performed using Statistical Product Service Solutions (SPSS, IBM Corporation) software 24.0, and *p* < 0.05 was considered to indicate statistical significance.

## Results

### YRHIP monitoring of ophthalmic follow-up

A total of 20,307 subjects were enrolled in the Yinzhou glaucoma screening program, which was conducted from November 26, 2020, to December 3, 2021. Based on the YRHIP, 344 patients were diagnosed with primary glaucoma, among whom 191 (55.5%) were diagnosed with PACD and 153 (44.5%) were diagnosed with POAG. Among them, 87 (45.5%, 87/191) PACD patients and 55 (35.9%, 55/153) POAG patients were identified as having newly diagnosed primary glaucoma, and 104 (54.5%, 104/191) PACD patients and 98 (64.1%, 98/153) POAG patients were identified as having previously diagnosed primary glaucoma (Figure 2).

### Sociodemographic and clinical characteristics of patients with previously diagnosed glaucoma and those with undiagnosed glaucoma

The mean age of patients with previously undiagnosed primary glaucoma, PACD or POAG was significantly lower than that of patients with previously diagnosed cases. The proportion of patients with PACS/PAC among those with previously undiagnosed PACDs (37.9%) was significantly greater than that among patients with previously diagnosed PACDs (12.5%). Similarly, the proportion of patients with NTG among those with previously undiagnosed POAG (50.9%) was significantly greater than that among those with previously diagnosed POAG (4.1%) (all *p* values<0.001). The proportions of patients with other ocular diseases among those with previously undiagnosed primary glaucoma (55.6%) and PACDs (52.9%) were significantly lower than those among those with previously diagnosed cases (73.3 and 72.1%, respectively) (all *p* values<0.01). The proportions of patients with primary glaucoma, PACDs or POAG significantly differed between the diagnosed and undiagnosed groups regarding the distribution of time since the last visit for eye care (all *p* values<0.001). The mean PVA of patients with previously undiagnosed primary glaucoma, PACDs or POAG was significantly better than that of patients with previously diagnosed cases (all *p* values<0.01). The mean IOP of patients with previously undiagnosed primary glaucoma or PACDs was significantly greater than that of patients with previously diagnosed cases (all *p* values<0.05). The mean VCDR of patients with previously undiagnosed primary glaucoma or PACDs was significantly smaller than that of patients with previously diagnosed glaucoma (all *p* values<0.001). The mean serum creatinine (SCr) and blood urea nitrogen (BUN) levels of patients with previously undiagnosed primary glaucoma or PACDs were significantly lower than those of patients with previously diagnosed cases (all *p* values <0.05). The mean total cholesterol (TC) and low-density lipoprotein (LDL) levels of patients with previously undiagnosed primary glaucoma or POAG were significantly higher than those of patients with previously diagnosed cases (all *p* values <0.05, Tables 2–4).

**Table 2:** Characteristics of patients with previously diagnosed versus undiagnosed primary glaucoma.

Characteristics	Previously Diagnosed *n* = 202	Previously undiagnosed *n* = 142	*p* value
Age (years), mean (SD)	72.0 ± 7.4	67.2 ± 6.6	**0.000**
Sex, *n* (%)			
Male	73 (36.1%)	46 (32.4%)	0.472
Female	129 (63.9%)	96 (67.6%)	
Married status, *n* (%)			
Unmarried/widowed	42 (20.8%)	31 (21.8%)	0.062
Married	137 (67.8%)	105 (73.9%)	
Unknown	23 (11.4%)	6 (4.2%)	
Screening communities, *n* (%)			
Urban area	134 (66.3%)	85 (59.9%)	0.219
Suburban area	68 (33.7%)	57 (40.1%)	
Diagnostic classification, *n* (%)			
PACD	104 (51.5%)	87 (61.3%)	0.072
POAG	98 (48.5%)	55 (38.7%)	
Other ocular diseases, *n* (%)			
No	54 (26.7%)	63 (44.4%)	**0.001**
Yes	148 (73.3%)	79 (55.6%)	
≥3 Systemic diseases, *n* (%)			
No	20 (9.9%)	23 (16.2%)	0.082
Yes	182 (90.1%)	119 (83.8%)	
Time since last visit for eye care *n* (%)			
<1 year	134 (66.3%)	44 (31.0%)	**0.000**
1 ~ 2 years	23 (11.4%)	17 (12.0%)	
2 + years	45 (22.3%)	81 (57.0%)	
BMI (kg/m^2^), mean (SD)*	23.42 ± 3.07	23.27 ± 2.85	0.648
MAP (mmHg), mean (SD)*	99.2 ± 9.2	99.6 ± 10.0	0.621
ASE (Dioptres), mean (SD)*	1.75 ± 2.80	1.61 ± 1.61	0.102
PVA (logMAR), mean (SD)	0.57 ± 0.58	0.34 ± 0.34	**0.000**
IOP (mmHg), mean (SD)*	14.8 ± 4.6	16.6 ± 6.0	**0.012**
VCDR, mean (SD)*	0.65 ± 0.21	0.55 ± 0.17	**0.000**
Fbg (mmol/L), mean (SD)*	5.90 ± 1.27	5.95 ± 1.63	0.964
Scr (umol/L), mean (SD)*	70.80 ± 21.12	65.07 ± 15.90	**0.026**
Bun (mmol/L), mean (SD)*	5.94 ± 4.01	5.18 ± 1.33	**0.011**
TC (mmol/L), mean (SD)*	4.72 ± 0.97	4.97 ± 1.13	**0.033**
TG (mmol/L), mean (SD)*	1.47 ± 0.76	1.57 ± 0.86	0.187
LDL (mmol/L), mean (SD)*	2.75 ± 0.79	2.96 ± 0.86	**0.023**
HDL (mmol/L), mean (SD)*	1.33 ± 0.32	1.40 ± 0.33	**0.033**

SD, standard deviation; PACD, primary angle closure disease; POAG, primary open angle glaucoma; BMI, body mass index; MAP, mean arterial pressure = 1/3(systolic pressure+2*diastolic pressure). ASE, absolute spherical equivalent; PVA, presenting visual acuity; logMAR, Logarithm of the Minimum Angle of Resolution; IOP, intraocular pressure; VCDR, vertical cup-to-disc ratio; Fbg, fasting blood glucose; Scr, serum creatinine; Bun, blood urea nitrogen; TC, total cholesterol; TG, triglyceride; LDL, low density lipoprotein; HDL, high density lipoprotein.

Systemic diseases included hypertension, diabetes mellitus, hyperlipoidemia, cardiovascular diseases, cerebrovascular diseases, arthritis, chronic kidney diseases, sleep disorders, depression and osteoporosis.

Other ocular diseases included keratopathy, cataract, retinopathy and maculopathy. Among them, retinopathy included diabetic retinopathy, retinal vascular oclusion, retinoschisis and other retinopathies, and maculopathy included age-related macular degeneration, epiretinal membrane, cystoid macular edema, high-myopia macular degeneration and macular hole.

*Missing data, including BMI (*n* = 2), MAP (*n* = 3), ASE (*n* = 97), IOP (*n* = 80), VCDR (*n* = 10), Fbg (*n* = 3), Scr (*n* = 7), Bun (*n* = 7), TC (*n* = 7), TG (*n* = 7), LDL (*n* = 6), and HDL (*n* = 6). Bold values indicate statistical significance (*p* < 0.05).

**Table 3:** Characteristics of patients with previously diagnosed versus undiagnosed primary angle closure diseases.

Characteristics	Previously diagnosed *n* = 104	Previously undiagnosed *n* = 87	*p* value
Age (years), mean (SD)	72.2 ± 6.8	67.3 ± 6.1	**0.000**
Sex, *n* (%)			
Male	30 (28.8%)	18 (20.7%)	0.196
Female	74 (71.2%)	69 (79.3%)	
Married status, *n* (%)			
Unmarried/widowed	25 (24.0%)	17 (19.5%)	**0.002**
Married	65 (62.5%)	69 (79.3%)	
Unknown	14 (13.5%)	1 (1.1%)	
Screening communities, *n* (%)			
Urban area	32 (30.8%)	36 (41.4%)	0.127
Suburban area	72 (69.2%)	51 (58.6%)	
Diagnostic classification, *n* (%)			
PACS/PAC	13 (12.5%)	33 (37.9%)	**0.000**
PACG	91 (87.5%)	54 (62.1%)	
Other ocular diseases, *n* (%)			
No	29 (27.9%)	41 (47.1%)	**0.006**
Yes	75 (72.1%)	46 (52.9%)	
≥3 Systemic diseases, *n* (%)			
No	17 (16.3%)	15 (17.2%)	0.869
Yes	87 (83.7%)	72 (82.8%)	
Time since last visit for eye care *n* (%)			
<1 year	66 (63.5%)	25 (28.7%)	**0.000**
1 ~ 2 years	11 (10.6%)	14 (16.1%)	
2 + years	27 (26.0%)	48 (55.2%)	
BMI (kg/m^2^), mean (SD)	23.26 ± 3.25	23.20 ± 2.82	0.890
MAP (mmHg), mean (SD)*	99.0 ± 9.7	99.0 ± 9.2	0.985
ASE (Dioptres), mean (SD)*	1.78 ± 2.83	1.69 ± 1.32	0.097
PVA (logMAR), mean (SD)	0.55 ± 0.55	0.36 ± 0.40	**0.003**
IOP (mmHg), mean (SD)*	14.2 ± 4.3	16.9 ± 5.5	**0.002**
VCDR, mean (SD)*	0.60 ± 0.21	0.49 ± 0.15	**0.000**
Fbg (mmol/L), mean (SD)*	5.85 ± 1.27	6.15 ± 1.92	0.434
Scr(umol/L), mean (SD)*	69.64 ± 21.52	61.32 ± 15.58	**0.004**
Bun (mmol/L), mean (SD)*	6.06 ± 5.06	4.99 ± 1.30	**0.006**
TC (mmol/L), mean (SD)*	4.82 ± 0.98	4.88 ± 1.22	0.678
TG (mmol/L), mean (SD)*	1.39 ± 0.75	1.56 ± 0.87	**0.041**
LDL (mmol/L), mean (SD) *	2.81 ± 0.79	2.90 ± 0.89	0.533
HDL (mmol/L), mean (SD)*	1.37 ± 0.33	1.42 ± 0.34	0.408

SD, standard deviation; PACS, primary angle closure disease; PAC, primary angle closure; PACG, primary angle closure glaucoma; BMI, body mass index; MAP, mean arterial pressure = 1/3(systolic pressure+2*diastolic pressure); ASE, absolute spherical equivalent; PVA, presenting visual acuity; logMAR, Logarithm of the Minimum Angle of Resolution; IOP, intraocular pressure; VCDR, vertical cup-to-disc ratio; Fbg, fasting blood glucose; Scr, serum creatinine; Bun, blood urea nitrogen; TC, total cholesterol; TG, triglyceride; LDL, low density lipoprotein; HDL, high density lipoprotein.

Systemic diseases included hypertension, diabetes mellitus, hyperlipoidemia, cardiovascular diseases, cerebrovascular diseases, arthritis, chronic kidney diseases, sleep disorders, depression and osteoporosis.

Other ocular diseases included keratopathy, cataract, retinopathy and maculopathy. Among them, retinopathy included diabetic retinopathy, retinal vascular oclusion, retinoschisis and other retinopathies, and maculopathy included age-related macular degeneration, epiretinal membrane, cystoid macular edema, high-myopia macular degeneration and macular hole.

*Missing data, including MAP (*n* = 1), ASE (*n* = 60), IOP (*n* = 46), VCDR (*n* = 9), Fbg (*n* = 1), Scr (*n* = 3), Bun (*n* = 4), TC (*n* = 3), TG (*n* = 3), LDL (*n* = 3), and HDL (*n* = 3). Bold values indicate statistical significance (*p* < 0.05).

**Table 4:** Characteristics of patients with previously diagnosed versus undiagnosed primary open angle glaucoma.

Characteristics	Previously Diagnosed *n* = 98	Previously undiagnosed *n* = 55	*p* value
Age (years), mean (SD)	71.8 ± 8.0	67.1 ± 7.3	**0.000**
Sex, *n* (%)			
Male	43 (43.9%)	28 (50.9%)	0.403
Female	55 (56.1%)	27 (49.1%)	
Married status, *n* (%)			
Unmarried/widowed	17 (17.3%)	14 (25.5%)	0.481
Married	72 (73.5%)	36 (65.5%)	
Unknown	9 (9.2%)	5 (9.1%)	
Screening communities, *n* (%)			
Urban area	36 (36.7%)	21 (38.2%)	0.859
Suburban area	62 (63.3%)	34 (61.8%)	
Diagnostic classification, *n* (%)			
HTG	94 (95.9%)	27 (49.1%)	**0.000**
NTG	4 (4.1%)	28 (50.9%)	
Other ocular diseases, *n* (%)			
No	25 (25.5%)	22 (40.0%)	0.062
Yes	73 (74.5%)	33 (60.0%)	
≥3 Systemic diseases, *n* (%)			
No	3 (3.1%)	8 (14.5%)	**0.018**
Yes	95 (96.9%)	47 (85.5%)	
Time since last visit for eye care *n* (%)			
<1 year	68 (69.4%)	19 (34.5%)	**0.000**
1 ~ 2 years	12 (12.2%)	3 (5.5%)	
2 + years	18 (18.4%)	33 (60.0%)	
BMI (kg/m^2^), mean (SD)*	23.59 ± 2.87	23.39 ± 2.93	0.676
MAP (mmHg), mean (SD)*	99.3 ± 8.5	100.5 ± 11.3	0.328
ASE (Dioptres), mean (SD)*	1.73 ± 2.81	1.50 ± 1.97	0.521
PVA (logMAR), mean (SD)	0.60 ± 0.61	0.31 ± 0.23	**0.000**
IOP (mmHg), mean (SD)*	15.4 ± 4.9	16.2 ± 6.7	0.705
VCDR, mean (SD)*	0.70 ± 0.20	0.65 ± 0.14	**0.062**
Fbg (mmol/L), mean (SD)*	5.94 ± 1.29	5.63 ± 0.92	0.319
Scr (umol/L), mean (SD)*	72.07 ± 20.72	70.97 ± 14.67	0.693
Bun (mmol/L), mean (SD)*	5.82 ± 2.45	5.48 ± 1.34	0.350
TC (mmol/L), mean (SD)*	4.61 ± 0.96	5.10 ± 0.97	**0.004**
TG (mmol/L), mean (SD)*	1.57 ± 0.77	1.58 ± 0.84	0.904
LDL (mmol/L), mean (SD)*	2.69 ± 0.80	3.04 ± 0.81	**0.011**
HDL (mmol/L), mean (SD)*	1.30 ± 0.31	1.39 ± 0.31	0.042

SD, standard deviation; HTG, high tension glaucoma; NTG, normal tension glaucoma; POAG, primary open angle glaucoma; BMI, body mass index; MAP, mean arterial pressure = 1/3(systolic pressure+2*diastolic pressure); ASE, absolute spherical equivalent; PVA, presenting visual acuity; logMAR, Logarithm of the Minimum Angle of Resolution; IOP, intraocular pressure; VCDR, vertical cup-to-disc ratio; Fbg, fasting blood glucose; Scr, serum creatinine; Bun, blood urea nitrogen; TC, total cholesterol; TG, triglyceride; LDL, low density lipoprotein; HDL, high density lipoprotein.

Systemic diseases included hypertension, diabetes mellitus, hyperlipoidemia, cardiovascular diseases, cerebrovascular diseases, arthritis, chronic kidney diseases, sleep disorders, depression and osteoporosis.

Other ocular diseases included keratopathy, cataract, retinopathy and maculopathy. Among them, retinopathy included diabetic retinopathy, retinal vascular oclusion, retinoschisis and other retinopathies, and maculopathy included age-related macular degeneration, epiretinal membrane, cystoid macular edema, high-myopia macular degeneration and macular hole.

*Missing data, including BMI (*n* = 2), MAP (*n* = 2), ASE (*n* = 37), IOP (*n* = 34), VCDR (*n* = 1), Fbg (*n* = 2), Scr (*n* = 4), Bun (*n* = 3), TC (*n* = 4), TG (*n* = 4), LDL (*n* = 3), and HDL (*n* = 3). Bold values indicate statistical significance (*p* < 0.05).

### Factors associated with previously undiagnosed primary glaucoma

According to the univariate logistic regression models, older age, worse PVA, larger VCDR, and higher SCr and BUN levels were associated with decreased odds of having previously undiagnosed primary glaucoma. Without other ocular diseases, increased time elapsed since the last visit for eye care and greater TC and LDL concentrations were associated with increased odds of having previously undiagnosed cases (all *p* values<0.05). After adjusting for covariates, older age was significantly associated with increased odds of having previously diagnosed primary glaucoma (OR 0.93, 95% CI 0.90–0.97; *p* < 0.01). Compared with patients whose last eye care visit was <1 year prior, patients whose last visit was >2 years prior were 5.3 times more likely to have previously undiagnosed cases (OR 5.34, 95% CI 2.97–9.60, *p* < 0.001). A worse PVA was associated with significantly greater odds of having previously diagnosed primary glaucoma (OR 0.37, 95% CI 0.18–0.76; *p* < 0.01; Table 5). According to the multivariable model, PACD patients who had last seen an eye care provider >1 year prior were more likely to have previously undiagnosed cases than those whose last visit was <1 year prior (all *p* values <0.05). A larger VCDR was significantly associated with decreased odds of previously undiagnosed PACDs (OR 0.12, 95% CI 0.02–0.99, *p* < 0.05; Table 6). Furthermore, older age was significantly associated with decreased odds of having previously undiagnosed POAG (OR 0.88, 95% CI 0.81–0.96; *p* < 0.01). Individuals with HTG had a significantly lower likelihood of having previously undiagnosed cases than those with NTG did (OR 0.02, 95% CI 0.00–0.09; *p* < 0.001). POAG patients who had last seen an eye care provider >2 years prior were more likely to have undiagnosed cases than were those who had their last visit <1 year prior (OR 7.20, 95% CI 2.32–22.29, *p* < 0.01; Table 7).

**Table 5:** Risk factors associated with previously-undiagnosed primary glaucoma.

Characteristics	Unadjusted OR	*p* value	Adjusted OR	*p* value
Age (years)	0.91 (0.88, 0.94)	**0.000**	0.93 (0.90, 0.97)	**0.001**
Sex, *n* (%)				
Male	0.85 (0.54, 1.33)	0.472		
Female	1			
Married status				
Unmarried/widowed	0.96 (0.57, 1.64)	0.889		
Married	1			
Screening communities				
Urban area	0.76 (0.49, 1.18)	0.219		
Suburban area	1			
Diagnostic classification				
PACD	1.49 (0.96, 2.31)	0.073		
POAG	1			
Other ocular diseases				
No	2.19 (1.39, 3.44)	**0.001**	0.91 (0.51, 1.63)	0.745
Yes	1			
≥3 Systemic diseases				
No	1.76 (0.93, 3.34)	0.085		
Yes	1			
Time since last visit for eye care				
<1 year	1		1	
1 ~ 2 years	2.25 (1.10, 4.59)	**0.026**	1.89 (0.85, 4.21)	0.117
2 + years	5.48 (3.33, 9.03)	**0.000**	5.34 (2.97, 9.60)	**0.000**
BMI (kg/m^2^)	0.98 (0.91, 1.06)	0.646		
MAP (mmHg)	1.01 (0.98, 1.03)	0.667		
ASE (Dioptres)	0.97 (0.87, 1.09)	0.620		
PVA (logMAR)	0.21 (0.10, 0.46)	**0.000**	0.37 (0.18, 0.76)	**0.007**
VCDR	0.08 (0.02, 0.25)	**0.000**	0.25 (0.06, 1.02)	0.053
Fbg (mmol/L)	1.03 (0.89, 1.19)	0.721		
Scr (umol/L)	0.98 (0.97, 1.00)	**0.009**	1.00 (0.98, 1.01)	0.581
Bun (mmol/L)	0.83 (0.71, 0.96)	**0.011**	0.88 (0.73, 1.06)	0.190
TC (mmol/L)	1.26 (1.02, 1.56)	**0.034**	0.96 (0.55, 1.68)	0.894
TG (mmol/L)	1.16 (0.88, 1.51)	0.291		
LDL (mmol/L)	1.36 (1.04, 1.77)	**0.024**	1.15 (0.57, 2.32)	0.699
HDL (mmol/L)	1.94 (1.00, 3.78)	0.052		

OR, odds ratio; PACD, primary angle closure disease; POAG, primary open angle glaucoma; BMI, body mass index; MAP, mean arterial pressure = 1/3(systolic pressure+2*diastolic pressure). ASE, absolute spherical equivalent; PVA, presenting visual acuity; logMAR, Logarithm of the Minimum Angle of Resolution; VCDR, vertical cup-to-disc ratio; Fbg, fasting blood glucose; Scr, serum creatinine; Bun, blood urea nitrogen; TC, total cholesterol; TG, triglyceride; LDL, low density lipoprotein; HDL, high density lipoprotein.

Systemic diseases included hypertension, diabetes mellitus, hyperlipoidemia, cardiovascular diseases, cerebrovascular diseases, arthritis, chronic kidney diseases, sleep disorders, depression and osteoporosis.

Other ocular diseases included keratopathy, cataract, retinopathy and maculopathy. Among them, retinopathy included diabetic retinopathy, retinal vascular oclusion, retinoschisis and other retinopathies, and maculopathy included age-related macular degeneration, epiretinal membrane, cystoid macular edema, high-myopia macular degeneration and macular hole. Bold values indicate statistical significance (*p* < 0.05).

**Table 6:** Risk factors associated with previously-undiagnosed primary angle closure diseases.

Characteristics	Unadjusted OR	*p* value	Adjusted OR	*p* value
Age (years)	0.89 (0.84, 0.93)	**0.000**	0.94 (0.88, 1.00)	0.058
Sex				
Male	0.64 (0.33, 1.26)	0.197		
Female	1			
Married status				
Unmarried/widowed	0.62 (0.32, 1.29)	0.214		
Married	1			
Screening communities				
Urban area	0.63 (0.35, 1.14)	0.128		
Suburban area	1			
Diagnostic classification				
PACS/PAC	4.28 (2.07, 8.83)	**0.000**	1.69 (0.71, 4.02)	0.234
PACG	1			
Other ocular diseases				
No	2.31 (1.26, 4.20)	**0.006**	1.24 (0.57, 2.69)	0.592
Yes	1			
≥3 Systemic diseases				
No	1.07 (0.50, 2.28)	0.869		
Yes	1			
Time since last visit for eye care				
<1 year	1		1	
1 ~ 2 years	3.36 (1.35, 8.28)	**0.009**	3.52 (1.19, 10.41)	**0.023**
2 + years	4.69 (2.43, 9.07)	**0.000**	3.18 (1.44, 7.00)	**0.004**
BMI (kg/m^2^)	0.99 (0.91, 1.09)	0.889		
MAP (mmHg)	1.00 (0.97, 1.03)	0.981		
ASE (Dioptres)	0.98 (0.83, 1.15)	0.783		
PVA (logMAR)	0.34 (0.15, 0.82)	**0.015**	0.68 (0.30, 1.54)	0.356
VCDR	0.04 (0.01, 0.24)	**0.000**	0.12 (0.02, 0.99)	**0.048**
Fbg (mmol/L)	1.13 (0.94, 1.36)	0.210		
Scr (umol/L)	0.97 (0.96, 0.99)	**0.006**	0.99 (0.96, 1.01)	0.310
Bun (mmol/L)	0.77 (0.63, 0.94)	**0.011**	0.82 (0.64, 1.06)	0.128
TC (mmol/L)	1.06 (0.81, 1.38)	0.677		
TG (mmol/L)	1.31 (0.91, 1.90)	0.149		
LDL (mmol/L)	1.15 (0.82, 1.63)	0.418		
HDL (mmol/L)	1.54 (0.65, 3.62)	0.328		

OR, odds ratio; PACS, primary angle closure disease; PAC, primary angle closure; PACG, primary angle closure glaucoma; BMI, body mass index; MAP, mean arterial pressure = 1/3(systolic pressure+2*diastolic pressure); ASE, absolute spherical equivalent; PVA, presenting visual acuity; logMAR, Logarithm of the Minimum Angle of Resolution; VCDR, vertical cup-to-disc ratio; Fbg, fasting blood glucose; Scr, serum creatinine; Bun, blood urea nitrogen; TC, total cholesterol; TG, triglyceride; LDL, low density lipoprotein; HDL, high density lipoprotein.

Systemic diseases included hypertension, diabetes mellitus, hyperlipoidemia, cardiovascular diseases, cerebrovascular diseases, arthritis, chronic kidney diseases, sleep disorders, depression and osteoporosis.

Other ocular diseases included keratopathy, cataract, retinopathy and maculopathy. Among them, retinopathy included diabetic retinopathy, retinal vascular oclusion, retinoschisis and other retinopathies, and maculopathy included age-related macular degeneration, epiretinal membrane, cystoid macular edema, high-myopia macular degeneration and macular hole. Bold values indicate statistical significance (*p* < 0.05).

**Table 7:** Risk factors associated with previously-undiagnosed primary open angle glaucoma.

Characteristics	Unadjusted OR	*p* value	Adjusted OR	*p* value
Age (years)	0.92 (0.88, 0.97)	**0.001**	0.88 (0.81, 0.96)	**0.003**
Sex				
Male	1.33 (0.68, 2.57)	0.403		
Female	1			
Married status				
Unmarried/widowed	1.58 (0.70, 3.56)	0.269		
Married	1			
Screening communities				
Urban area	0.94 (0.48, 1.86)	0.859		
Suburban area	1			
Diagnostic classification				
HTG	0.04 (0.01, 0.13)	**0.000**	0.02 (0.00, 0.09)	**0.000**
NTG	1			
Other ocular diseases				
No	1.95 (0.96, 3.94)	0.064		
Yes	1			
≥3 Systemic diseases				
No	5.39 (1.37, 21.26)	**0.016**	4.60 (0.67, 31.81)	0.122
Yes	1		1	
Time since last visit for eye care				
<1 year	1		1	
1 ~ 2 years	0.90 (0.23, 3.50)	0.873	0.42 (0.07, 2.49)	0.337
2 + years	6.56 (3.05, 14.13)	**0.000**	7.20 (2.32, 22.29)	**0.001**
BMI (kg/m^2^)	0.98 (0.87, 1.10)	0.674		
MAP (mmHg)	1.01 (0.98, 1.05)	0.437		
ASE (Dioptres)	0.96 (0.83, 1.13)	0.634		
PVA (logMAR)	0.09 (0.02, 0.36)	**0.001**	0.22 (0.04, 1.19)	0.078
VCDR	0.20 (0.03, 1.28)	0.090		
Fbg (mmol/L)	0.78 (0.57, 1.07)	0.123		
Scr (umol/L)	1.00 (0.98, 1.02)	0.730		
Bun (mmol/L)	0.91 (0.75, 1.11)	0.357		
TC (mmol/L)	1.68 (1.17, 2.41)	**0.005**	2.23 (0.68, 7.32)	0.187
TG (mmol/L)	1.02 (0.67, 1.55)	0.938		
LDL (mmol/L)	1.71 (1.12, 2.61)	**0.013**	0.62 (0.16, 2.42)	0.493
HDL (mmol/L)	2.49 (0.85, 7.29)	0.096		

OR, odds ratio; HTG, high tension glaucoma; NTG, normal tension glaucoma; POAG, primary open angle glaucoma; BMI, body mass index; MAP, mean arterial pressure = 1/3(systolic pressure+2*diastolic pressure); ASE, absolute spherical equivalent; PVA, presenting visual acuity; logMAR, Logarithm of the Minimum Angle of Resolution; VCDR, vertical cup-to-disc ratio; Fbg, fasting blood glucose; Scr, serum creatinine; Bun, blood urea nitrogen; TC, total cholesterol; TG, triglyceride; LDL, low density lipoprotein; HDL, high density lipoprotein.

Systemic diseases included hypertension, diabetes mellitus, hyperlipoidemia, cardiovascular diseases, cerebrovascular diseases, arthritis, chronic kidney diseases, sleep disorders, depression and osteoporosis.

Other ocular diseases included keratopathy, cataract, retinopathy and maculopathy. Among them, retinopathy included diabetic retinopathy, retinal vascular oclusion, retinoschisis and other retinopathies, and maculopathy included age-related macular degeneration, epiretinal membrane, cystoid macular edema, high-myopia macular degeneration and macular hole. Bold values indicate statistical significance (*p* < 0.05).

## Discussion

This was the first study to analyze risk factors associated with previously undiagnosed PACDs and POAG using regional health big data from all levels of hospitals in the Yinzhou district, China. Previously diagnosed and undiagnosed glaucoma was identified through EMRs, which differs from the methods used in other population-based studies involving interviewer-administered questionnaires. Patty et al. (33) reported that self-reports underestimate disease burden, with a 37.7% sensitivity for glaucoma. Our study utilized medical records, effectively preventing patient recall bias. These results will contribute to the optimization of screening programs and the achievement of higher case confirmation rates in community settings.

Individuals with previously diagnosed primary glaucoma, PACDs and POAG were significantly older than undiagnosed patients were, and older age was associated with a lower risk of being undiagnosed. This might be because glaucoma progresses with age, and the disease is more symptomatic in older individuals; in addition, the prevalence of glaucoma increases with age (1, 13, 21). Moreover, in this study, the mean PVA in the previously diagnosed group was significantly worse than that in the undiagnosed group. For individuals with visual damage, eye care providers might be more proactive in conducting comprehensive ophthalmic examinations (34), including visual field testing and optic disc imaging, which is conducive to the early detection and diagnosis of glaucoma. Furthermore, the proportions of patients with ocular conditions and **≥** 3 systemic comorbidities in the previously diagnosed group were significantly greater than those in the undiagnosed group. We speculated that individuals from the previously diagnosed group had more frequent visits for medical care and thus could be more likely to be tested for glaucoma (35). The proportion of patients whose most recent eye care visit was less than 1 year prior was also significantly greater in the diagnosed group than that in the undiagnosed group, indicating that individuals with a previous diagnosis might have greater awareness of the need for eye care and subsequent eye care-seeking behaviors (35). The mean VCDR of previously diagnosed glaucoma patients was greater than that of undiagnosed glaucoma patients, indicating that previously diagnosed glaucoma patients had more visual damage than undiagnosed glaucoma patients did, consistent with results in Japan (20, 36), Korea (17), Greece (37) and the EPIC-Norfolk Eye Study (18).

We found that increased time since the last visit for eye care was significantly associated with a greater likelihood of undiagnosed primary glaucoma, PACDs, and POAG. These results were consistent with the findings of other epidemiological reports (15, 17, 37, 38). A lack of regular eye care utilization decreases the likelihood of early glaucoma detection. The use of ophthalmic resources may be challenging due to the lack of awareness of the irreversibility of glaucoma, the cost of eye examination, poor access to eye care, conflicting outpatient arrangements, and a lack of medical insurance. Poor adherence may increase the risk of developing irreversible blindness due to undetected glaucoma. Furthermore, eye care utilization patterns are related to glaucoma unawareness (19, 35). The awareness of glaucoma among patients who consulted private ophthalmologists was greater than that among those who received eye care from opticians/optometrists (35). Compared with receiving care from optometrists alone, receiving care from an ophthalmologist alone or from both ophthalmologists and optometrists was significantly associated with POAG detection (19, 35).

Among patients with primary glaucoma, a worse PVA was less likely to be observed in previously diagnosed patients than in previously undiagnosed patients, even after adjustment for potential confounding factors. We found that the mean PVA in the diagnosed group was significantly worse than that in the undiagnosed group in this study, and these results are inconsistent with those of previous population-based studies (16, 17, 39). The disparity between these findings indicated that most individuals in China did not seek eye care until they exhibited obvious visual symptoms. Unequal access to eye care and a lack of population-based screening programs increase the likelihood of advanced glaucoma (40, 41).

Patients with HTG were approximately 98% less likely to be previously undiagnosed than patients with NTG were. We hypothesized that considering IOP as a vital risk factor for glaucoma (42), eye care providers would be diligent in the clinical assessment and diagnosis of glaucoma. Although pretreatment IOP data for diagnosed patients were unavailable, HTG patients had significantly greater IOP and needed more frequent visits for eye care. These individuals may be diagnosed with glaucoma at an early stage. However, compared with patients with HTG, patients with NTG are less likely to be diagnosed early due to a lower baseline IOP, a chronic course, insufficient recognition of NTG and inability to screen for NTG among ophthalmologists (43). A low frequency of seeking eye care among patients and a severe underestimation of the prevalence of NTG in clinical practice can negatively impact the diagnosis of NTG (10, 11, 44).

A larger VCDR was associated with a reduced risk of being previously undiagnosed with PACD, which is consistent with the findings of prior studies. Topouzis et al. (37) and Iwase et al. (36) reported that a smaller VCDR correlated with a higher risk of undiagnosed POAG, whereas Kumejima (20) reported that better mean deviation and the absence of signs indicative of prior acute angle closure contributed to previously undiagnosed PACG. Collectively, these findings underscore that specific anatomical and functional markers may modulate glaucoma detection. In China, nationwide cataract surgery outreach programs have been extensively implemented to mitigate cataract-related blindness (45, 46), and our data revealed a potential association between this initiative and PACD diagnosis: 30.8% (32/104) of patients with a confirmed PACD diagnosis had a history of cataract surgery, whereas only 3.4% (3/87) of individuals who underwent cataract surgery had previously undiagnosed PACD. We hypothesize that this discrepancy arises because anterior segment examinations (prerequisites for cataract surgery) facilitate PACD detection, potentially increasing diagnosis rates via increased surgical access. However, our retrospective data limited the inclusion of anterior segment quantitative parameters in the regression models.

There were several limitations in this study. First, we did not conduct visual field testing in primary health care centers, as this was not feasible in a large-scale community-based screening with a high population density. Therefore, we could not analyze the sensitivity and specificity of this glaucoma screening program or assess the exact prevalence of undiagnosed glaucoma. Although we identified glaucoma using strict definitions and our data covered all levels of hospitals in the Yinzhou district, the factors associated with previously undiagnosed glaucoma might be more generalizable to routine clinical practice. Second, data on pretreatment IOP, mean deviation, and eye care utilization patterns were not available; thus, we could not adjust for these variables. The diagnostic classification of previously diagnosed glaucoma patients was in accordance with the ICD-10 or diagnostic categories from EMRs, and we cannot guarantee that all patients were correctly classified. However, the data used in this study should be more accurate than those based on self-report questionnaires. Third, we did not calculate the cost of identifying glaucoma or GS patients, as that information is not the focus of our study.

## Conclusion

In our study, a younger age, a longer duration since the last visit for eye care, a smaller VCDR, and NTG were associated with a greater likelihood of glaucoma being previously undiagnosed. Additionally, these risk factors for previously undiagnosed primary glaucoma highlight the need to increase glaucoma awareness and the utilization of ophthalmic resources.

## Data Availability

The raw data supporting the conclusions of this article will be made available by the authors, without undue reservation.

## Footnotes

1
http://www.medresman.org.cn/

## Glossary

YRHIP: Yinzhou regional health information platform
PVA: Presenting visual acuity
VCDR: Vertical cup-to-disc ratio
HTG: High-tension glaucoma
POAG: Primary open angle glaucoma
PACG: Primary angle closure glaucoma
RCT: randomized controlled trial
IOP: Intraocular pressure
OCT: Optical coherence tomography
GS: Glaucoma suspect
PACD: Primary angle closure diseases
PACS: Primary angle closure suspect
PAC: Primary angle closure
GON: Glaucomatous optic neuropathy
PAS: Peripheral anterior synechiae
NTG: Normal-tension glaucoma
EMRs: Electronic medical records
EHRs: electronic health records
ICD-10: International Classification of Diseases 10th edition
BMI: Body mass index
MAP: Mean arterial pressure
ASE: Absolute spherical equivalent
Scr: Serum creatinine
Bun: Blood urea nitrogen
TC: total cholesterol
LDL: low density lipoprotein
NMFP: nonmydriatic fundus photography
MAP: mean arterial pressure
logMAR: Logarithm of the Minimum Angle of Resolution
Fbg: Fasting blood glucose
TG: Triglyceride
HDL: High density lipoprotein
